# Stabilized Supralinear Network Model of Responses to Surround Stimuli in Primary Visual Cortex

**DOI:** 10.1523/ENEURO.0459-24.2025

**Published:** 2025-05-13

**Authors:** Dina Obeid, Kenneth D. Miller

**Affiliations:** ^1^Center for Theoretical Neuroscience and Swartz Program in Theoretical Neuroscience, College of Physicians and Surgeons and Mortimer B. Zuckerman Mind Brain Behavior Institute, Columbia University, New York City, NY 10027; ^2^Harvard John A. Paulson School Of Engineering And Applied Sciences, Harvard University, Cambridge, MA 02138; ^3^Department of Neuroscience and Kavli Institute for Brain Science, College of Physicians and Surgeons and Mortimer B. Zuckerman Mind Brain Behavior Institute, Columbia University, New York City, NY 10027

## Abstract

In the mammalian primary visual cortex (V1), there are complex interactions between responses to stimuli present in the cell’s classical receptive field (CRF) or “center” and in the surrounding region or “surround.” The circuit mechanisms underlying these behaviors are likely to represent more general cortical mechanisms for integrating information. Here, we develop a circuit model that accounts for three important features of surround suppression (suppression of response to a center stimulus by addition of a surround stimulus): (1) The surround stimulus suppresses the inhibitory and excitatory currents that the cell receives; (2) The strongest suppression arises when the surround orientation matches that of the center stimulus, even when the center stimulus orientation differs from the cell’s preferred orientation; and (3) A surround stimulus of a given orientation most strongly suppresses that orientation’s component of the response to a plaid center stimulus (“feature-specific suppression”). We show that a stabilized supralinear network (SSN) with biologically plausible connectivity and synaptic efficacies that depend on cortical distance and orientation difference between units can consistently reproduce phenomena (1) and (3), and, qualitatively, phenomenon (2). We explain the mechanism behind each result. We argue that phenomena (2) and (3) are independent: the model with some aspects of connectivity removed still produces phenomenon (3) but not (2). The model reproduces the rapid time scale of activity decay observed in mouse V1 when thalamic input to V1 is silenced. Finally, we show that these results hold both in networks with rate-based and conductance-based spiking units.

## Significance Statement

A visual neuron responds to stimuli present in its classical receptive field or “center.” These responses are modulated in a complex manner by stimuli outside the receptive field i.e., in “the surround.” Understanding the underlying circuit behind center-surround interactions is crucial to understanding fundamental brain computations. Here, we focus on a set of key center-surround phenomena in the primary visual cortex. We demonstrate how complex aspects of cortical computation can be carried out by the local cortical circuit. We demonstrate that the SSN, a mechanism that accounts for a multitude of cortical response properties, can also account for these phenomena, given appropriate connectivity. We also demonstrate that this mechanism can be achieved in a biologically realistic spiking network.

## Introduction

Electrophysiological recordings from cells in the primary visual cortex (V1) reveal that visual stimuli presented outside the CRF of a neuron (the surround) can modulate the neuron’s response to a stimulus present in its CRF (center) in complex ways. The degree and direction of modulation depends on the distance between the center and surround, the contrasts of the stimuli, their relative orientations, etc. (e.g., Sillito et al., 1995; Sceniak et al., 1999; Akasaki et al., 2002; Cavanaugh et al., 2002; Bair et al., 2003; Shen et al., 2007; Wang et al., 2009). Understanding the underlying circuit mechanisms is crucial to understanding fundamental brain computations.

To address these mechanisms, we build a spatially extended, biologically constrained model of layer 2/3 of V1 of animals with orientation maps. We investigate the conditions on the connectivity and synaptic efficacies of the model circuit that allow it to generate a number of key properties of the V1 circuit, focusing on surround suppression, the suppression of response to a center stimulus by addition of a surround stimulus (Angelucci et al., 2017). We assume that the surround influence is carried by V1 lateral connections. However, projections from V1 to higher visual areas and back could also contribute (Nassi et al., 2013; Angelucci et al., 2017); the conditions we define on the connectivity must be satisfied by the net lateral influence via these two routes.

We first show that our model can successfully reproduce surround suppression in similar strength and with similar contrast dependence to that observed in layer 2/3 of V1 of animals with orientation maps, as has been shown previously in a similar model (Rubin et al., 2015). We then address three additional aspects of surround suppression: (1) Surround suppression is accompanied by a decrease in the inhibition as well as the excitation that a cell receives (Ozeki et al., 2009; Adesnik, 2017); (2) The strongest suppression arises when the orientation of the surround stimulus matches that of the center stimulus, even when the center orientation is not optimal for the cell (Sillito et al., 1995; Shen et al., 2007; Shushruth et al., 2012; Trott and Born, 2015); and (3) A surround stimulus with orientation matching the orientation of one component of a plaid center stimulus more strongly suppresses the response of the matching component (Trott and Born, 2015). We find that to match (2), local connectivity, in addition to being strong, must be broadly tuned for orientation; however, to match (3), this additional requirement is not needed. That is, effect (3) can occur without effect (2). This argues against the proposal that effects (2) and (3) are two manifestations of a single mechanism (Trott and Born, 2015).

It may appear that property (1) has been addressed previously. It was shown in a simplified model to require that the excitatory subnetwork be unstable by itself, but be stabilized by feedback inhibition, thus constituting an *inhibition-stabilized network* (Ozeki et al., 2009). However, the conditions for this to arise in a spatially extended circuit must be worked out. Inhibition received was shown to be decreased by surround suppression in a spatially one-dimensional model in Rubin et al. (2015). That paper also presented a spatially two-dimensional model, but inadvertently did not examine this property in that model. We have since found that the inhibition received by cells was increased by surround suppression in that 2-D model. Here, we determine the conditions needed for inhibition received to decrease in a spatially two-dimensional model. We find that this requires local connectivity strong enough that the local network is stabilized by feedback inhibition. Since the local density of the cells on the grid is much less than the cell density in biological networks, local connection strength on the grid must be especially strong to achieve this behavior, as we explain in more detail later in the paper.

The above results focus on steady state activity. We also show that our model is consistent with a key result on V1 dynamics: when the thalamic input to V1 is abruptly silenced, V1 activity decays very quickly, with a decay time constant of about 10 ms (Reinhold et al., 2015). A similar result was obtained when thalamic input to a frontal cortical area, ALM, was silenced (Guo et al., 2017), suggesting this rapid decay is a more general property of cortical circuitry. This was surprising both because the rise and decay times of responses to visual stimuli ranged from hundreds of milliseconds to seconds, and because the strong recurrent excitatory loops often assumed for V1 and other cortical areas will typically lead to slow decay timescales (although this effect can be cancelled by balancing feedback inhibition) (Murphy and Miller, 2009). For example, the ring model of orientation selectivity produces decay time constants ranging from 50 ms in the more weakly coupled, “homogeneous” regime to many seconds in the more strongly coupled, “marginal” or attractor regime (Goldberg et al., 2004). Given the strong local recurrent excitation required in our model, it is important to show that the model replicates the fast decay dynamics observed in V1.

Finally, we show that our results hold in networks with conductance-based spiking units. This demonstrates that similar mechanisms will operate in the more biological context of a spiking neural network (see also Sanzeni et al., 2020a, 2020b).

## Model

### Model overview

To investigate the computational role of V1 lateral connections, we build a 2-dimensional spatially extended model of layer 2/3 of the primary visual cortex of animals with orientation maps. Retinotopic position changes smoothly across both spatial dimensions, while preferred orientation of neurons is determined by their position in the orientation map. The Cortical Magnification Factor (CMF), which expresses how many mm of cortex represents one degree in visual angle, constrains the size of a neuron’s receptive field (RF), as we describe below. The connectivity in the model is broadly constrained by biological data. Neurons in V1 layer 2/3 are found to form dense axonal projections at distances of a few hundred μm, and sparse long range horizontal projections that target cells of similar orientation preferences. These long range connections, which can reach up to 3 mm in cat and 10 mm in monkey, arise from excitatory cells, and give rise to the patchy connectivity observed in V1 (Amir et al., 1993; Bosking et al., 1997; Stettler et al., 2002). In comparison, inhibitory cells primarily form short-range connections.

We first present results from a rate-based model. The units in the rate-based model are taken to have an expansive or supralinear, power-law transfer function (Albrecht and Hamilton, 1982; Albrecht, 1991; Heeger, 1992; Carandini et al., 1997, 1999; Hansel and van Vreeswijk, 2002; Miller and Troyer, 2002; Finn et al., 2007), as expected for neurons whose spiking is driven by input fluctuations rather than by the mean input (Hansel and van Vreeswijk, 2002; Miller and Troyer, 2002). Rubin et al. (2015) and Ahmadian et al. (2013) showed that when neural-like units have such a power-law transfer function, responses with nonlinear behaviors observed in visual cortex emerge due to network dynamics. The authors called this mechanism the stabilized supralinear network (SSN). They showed that the SSN mechanism can explain normalization and surround suppression and their nonlinear dependencies on stimulus contrast, which are observed across multiple sensory cortical areas.

To verify that our results are robust and independent of the neuron model, we also build a conductance-based spiking neural network model, and show that all our key results still hold.

While we have attempted to keep the model as simple as possible, the large-scale simulations conducted here necessarily have a large number of parameters. We did extensive exploration to find determinants of model behavior, far more than we can present, in order to arrive at the actual parameter choices and simulations we do present. However, it is impossible to completely search the parameter space, so the conclusions we draw from our explorations can only be tentative. As we present the structure of the model, parameter choices, and determinants of model behavior, we explain to the reader the considerations that guide our choices and conclusions. These explanations are necessarily presented in somewhat subjective terms, such as “it seems” and “we think,” as is appropriate for the tentative nature of our conclusions from explorations.

### Model details

We use a grid of 75 × 75 grid points. We place one excitatory cell (E), and one inhibitory cell (I) at each location on the lattice, and thus have 5,625 E cells and 5,625 I cells in the network. Use of a more realistic ratio of E to I cells (e.g., 80/20 instead of 50/50) should not affect results, so long as the density of cells remains sufficient that each small iso-oriented region contains I cells; having fewer I cells in a given region can be compensated by increasing the strengths of their projections. Because we must, for practical reasons, simulate on a discrete grid that is far less dense than the density of cortical cells, we use a 50/50 ratio to ensure that no local region is lacking in I cells. In unpublished work, we studied SSN behavior in spiking networks consisting of 1,152 E cells and 288 I cells (E/I ratio of 80/20), in models in which the units were characterized by either their location (considering a group of units with the same preferred orientation) or preferred orientation (considering a group of units at the same location). We found that the behavior of these networks was consistent with SSN predictions in the parameter regime we studied ( i.e., with Ω_*E*_ < Ω_*I*_ < 0, using parameters defined in Ahmadian et al., 2013, see below for more details).

We take the map to represent 16 × 16 degrees of visual space, with position in visual space varying linearly across the map, and assume a Cortical Magnification Factor (CMF) of 0.5 mm/deg. Thus the grid represents 8.0 × 8.0 mm of cortex, with each grid interval representing 0.213 degrees and 107 μm of cortical distance. We use periodic boundary conditions; our results are independent of that condition. This is verified by removing periodic boundary conditions, and adjusting the weight efficacy matrix to compensate for the lost connections.

**Figure 1.**
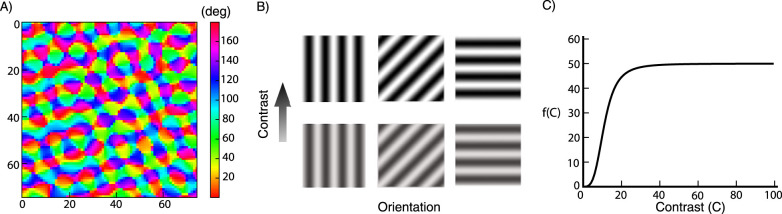
***A***, Orientation map over the 75 × 75 grid of cells (numbers on axes indicate position on the grid). The color corresponds to the preferred orientation of the cells at a given location. ***B***, Gratings with different orientations and contrasts. ***C***, External input as a function of the stimulus contrast (Eq. 3).

We superpose on the grid an orientation map, specifying the preferred orientations of cells at the corresponding grid points (Fig. 1*A*). The orientation map is generated randomly using the method described in Kaschube et al. (2010) (their supplementary materials, Eq. (20)). To summarize, we superpose *n* complex plane waves to form a function *z*(***x***) of two-dimensional spatial position **x**:

$$z(x)=\sum_{\;j=1}^{n}e^{i(l_{j}k_{j}\cdot x+\phi_{j})}.$$

Here, $k_{j}=k(\cos\;(\;j\pi /n),\sin\;(\;j\pi /n))$, with signs *l*_*j*_ ∈ { + 1, − 1} and phases *ϕ*_*j*_ ∈ [0, 2*π*) randomly chosen. Writing *z*(**x**) = *r*(**x**)*e*^*i*Φ(**x**)^ for real amplitude *r*(**x**) and phase Φ(**x**), we take the preferred orientation at each grid point **x** to be Φ(**x**)/2. We use a map spatial frequency of $k=\frac{8\;cycles}{75\;gridpoints}$, i.e., a map with on average 8 full periods of the orientation map across the length or width of the grid, and *n* = 30. The orientation map is not periodic, so there is a discontinuity in orientation at the grid borders, although the retinotopy and intracortical connections wrap around. In our results, we report on cells sampled away from the boundary (20 < *x* < 60, 20 < *y* < 60, in terms of the grid coordinates that go from 1 to 75 in each dimension) to avoid boundary effects.

The excitatory cells form both short- and long-range connections, while the inhibitory cells form only short-range connections. The connection strength from a unit of type *Y* at grid location *b* to a unit of type *X* at grid location *a*, *X*, *Y* ∈ {*E*, *I*}, is written $W_{XY}^{ab}$. Let the units at *a* and *b* have positions **x**_*a*_ and **x**_*b*_, respectively, and preferred orientations *θ*_*a*_ and *θ*_*b*_. Here and below we define the distance between two spatial positions, |**x**_*a*_ − **x**_*b*_|, to be the shortest distance across the grid given periodic boundary conditions, and the distance between two orientations, |*θ*_*a*_ − *θ*_*b*_|, to be the shortest angular distance between them around the $180^{\circ }$ circle of orientations. The connection strength is given by $W_{XY}^{ab}=J_{XY}p_{XY}(|x_{a}-x_{b}|)q_{XY}(|\theta_{a}-\theta_{b}|)$. Here, *p*_*XY*_(*x*) describes the dependence of strength on the spatial distance between the units, while *q*_*XY*_(*θ*) describes the dependence on the difference between their preferred orientations, and *J*_*XY*_ is a parameter that sets the strength of the connections. The function *p*_*XY*_(*x*) is specified as follows. For projections of excitatory cells, *p*_*XE*_(*x*) is 1 for distances *x* ≤ *L*_*o*_, and then decays as a Gaussian with standard deviation *σ*_*XE*_. *L*_*o*_ = 3 grid intervals (321 μm), *σ*_*EE*_ = 3 grid intervals (321 μm), and *σ*_*IE*_ = 6 grid intervals (642 μm). For projections of inhibitory cells, *p*_*XI*_(*x*) is Gaussian with standard deviation *σ*_*EI*_ = *σ*_*II*_ = 2 grid intervals (214 μm).

**Figure 2.**
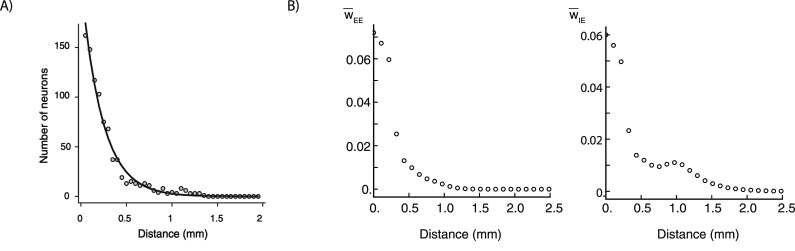
***A***, Density of retrogradely labeled excitatory cells in macaque V1 vs. distance from the retrograde tracer injection site in V1. This is proportional to the probability that a neuron at one site in V1 projects to another V1 site, as a function of the distance between them. The line is an exponential fit to the data, with a length constant of 230 μm. Reproduced with permission from Markov et al. (2011). ***B***. For the connection strengths used in the model, we plot the average strength of a weight between two cells a given distance apart, averaged over all cell pairs, for a connection from an excitatory cell to an excitatory cell (*W*_*EE*_, left) or to an inhibitory cell (*W*_*IE*_, right). This reflects both the explicit dependence on distance, *p*_*XE*_(|**x**_*a*_ − **x**_*b*_|), and the dependence *q*_*XE*_(|*θ*_*a*_ − *θ*_*b*_|) on preferred orientation difference along with the orientation map (Fig. 1*A*) (the functions *p* and *q* are defined in the text). The bump in the right panel arises because *E* → *I* long range connections have a Gaussian spatial profile that remains nonzero around 1 mm, where the neuron’s preferred orientation tends to recur since this is roughly the period of the orientation map.

The reasons for these choices for the connectivity structure and parameter values are as follows. The extra-strong local connectivity of E cells (no falloff with distance within *L*_*o*_) is used to compensate for our finite grid, which has spacing of 107 μm between neighboring cells. This low density of cells makes it difficult to capture the strong local connectivity of cortex. We found that we needed strong, though not flat, local connectivity for the inhibition received by cells to decrease with surround suppression. In addition, for the most suppressive surround to have orientation matching the center stimulus orientation regardless of a cell’s preferred orientation, we needed such strong local connectivity between cells with different preferred orientations, which are some distance apart due to the orientation map. This required us to make the strong connectivity locally flat with distance (within the distance *L*_*o*_). With a more realistic density we don’t think this would be necessary. For inhibition to decrease with surround suppression, we also seem to need the E→I connectivity to become stronger relative to the E→E connectivity with increasing distance (hence the choice of *σ*_*IE*_ larger than *σ*_*EE*_); there is little data on this issue, so this represents a prediction of the model. As will be shown below (see Fig. 2), when combined with the orientation dependence of weights, these parameters produce an empirical falloff of excitatory connection strength with distance that reasonably agrees with experiments. The smaller *σ*’s of the inhibitory connections model the inhibitory cells making only short-range, not long-range connections.

We choose the *J*_*XY*_, which in turn determine the *W*_*XY*_ as described above, to satisfy two conditions. First, we require that *W*_*II*_ − *W*_*EI*_ < 0. Here, *W*_*XY*_ is the mean, across cells of type *X*, of the total synaptic weight from units of type Y to a unit of type X. We do this to set Ω_*E*_ < 0, where Ω_*E*_ is a parameter defined in Ahmadian et al. (2013); for equal external inputs to the excitatory and inhibitory cells as we use here, Ω_*E*_ = *W*_*II*_ − *W*_*EI*_. The significance of setting Ω_*E*_ < 0 is as follows: as shown by Ahmadian et al. (2013), for Ω_*E*_ < 0, as external input grows from zero, excitatory responses first grow supralinearly, but their growth then becomes sublinear and saturates (and ultimately would supersaturate) for increasingly strong external input. For Ω_*E*_ > 0, excitatory responses instead go from growing supralinearly to growing linearly with increasing external input. In the Ω_*E*_ < 0 regime, it was also important that Ω_*E*_ < Ω_*I*_, where for equal inputs Ω_*I*_ = *W*_*IE*_ − *W*_*EE*_. For our parameters, Ω_*E*_ = −0.49 and Ω_*I*_ = 3.59. We did not explore the Ω_*E*_ > 0 regime and so do not know if being in the Ω_*E*_ < 0 regime is necessary for our results. The second condition on the *J*_*XY*_ was that, with increasing stimulus size, the loss of excitatory input to inhibitory cells from nearby surround-suppressed excitatory cells is greater than their gain in excitatory input from far away excitatory cells, which is necessary for the net inhibition received by excitatory cells to decrease with surround suppression.

For all cells regardless of pre- or postsynaptic type, the function *q*_*XY*_(*θ*) has the form of a Gaussian with a non-zero baseline: $q_{XY}(\theta )=A_{XY}+B_{XY}e^{\frac{-\theta^{2}}{2*(\sigma_{XY}^{\;ori}{)}^{2}}}$. For projections of I cells and of E cells at distances less than *L*_*o*_, *A*_*XI*_ = *A*_*XE*_ = 0.2, *B*_*XI*_ = *B*_*XE*_ = 0.8 and $\sigma_{XI}^{\;ori}=\sigma_{XE}^{\;ori}=55^{\circ }$. For projections of excitatory cells at distances greater than *L*_*o*_, *A*_*XE*_ = 0.14, *B*_*XE*_ = 0.86 and $\sigma_{XE}^{\;ori}=25^{\circ }$. The constants *J*_*XY*_ are, for I projections, *J*_*EI*_ = 0.0528 and *J*_*II*_ = 0.0288; for E projections, at distances less than *L*_*o*_, *J*_*EE*_ = 0.072 and *J*_*IE*_ = 0.06, while at distances greater than *L*_*o*_, *J*_*EE*_ = *J*_*IE*_ = 0.036. These specific values of *J*_*XY*_ arose in our simulations in the course of trying to get biologically realistic values of firing rates and suppression indices, and they were inadvertently kept unrounded in the final simulations. We nevertheless expect that rounding them to two significant digits would cause little or no visible changes and even to one digit should not cause qualitative changes. Any values that satisfy the qualitative conditions on the J’s described above should give the same qualitative results. The qualitative idea that at short distances connections are broad in orientation space, while at larger distances they are more tightly tuned, matches measurements of excitatory axonal projections in monkey V1 (Stettler et al., 2002) and of excitatory synaptic input in ferret (Wilson et al., 2016). Since we don’t have quantitative data on the local orientation tuning width of the excitatory connections, *σ*^*ori*^, from these species, we estimated this from mouse V1 data (Ko et al., 2013), $\sigma^{\;ori}=55^{\circ }$. We have found that making these connections considerably wider does not qualitatively change our results.

The combination of distance dependence and orientation dependence, along with the orientation map, yields an empirical dependence of excitatory connection strengths on distance that reasonably matches the density of retrogradely labelled excitatory cells at different distances observed in macaque V1 by Markov et al. (2011) (Fig. 2). The major discrepancy is the “bump” in E→I connectivity strength centered around 1 mm, which results from the particular way we implemented the more general requirement that E→I connectivity should become stronger relative to the E→E connectivity with increasing distance. Since synaptic strengths vs. distance have not been measured, this requirement remains a prediction of the model.

Note that the connectivity is completely deterministic, so that all heterogeneity in the responses of different cells comes from their having different positions in the orientation map and therefore receiving different inputs.

To define the visual stimuli, we ignore stimulus features like spatial frequency and phase, and consider only three features: contrast, orientation and size (Fig. 1*B*). The cells in the model behave like ideal complex cells, in that their external input induced by a drifting grating is static in time. The external input to a neuron located at position **x_o_** = (*x*_*o*_, *y*_*o*_) with preferred orientation *θ*_*o*_, from a square stimulus of contrast C, orientation *θ*_*s*_ and sides of length ℓ degrees that is centered at **x_s_** = (*x*_*s*_, *y*_*s*_) (with zero contrast outside the square), is given by

$$\Sigma_{C\theta_{s}}(x_{o})=f(C)h_{\ell }(x_{s}-x_{o})g(|\theta_{s}-\theta_{o}|).$$

Here, *f*(*C*) is a Naka-Rushton function given by

$$f(C)=\frac{\;f_{\max}\;*\;C^{3.5}}{C_{50}^{3.5}+C^{3.5}}$$

with $f_{\max}=50$ and *C*_50_ = 11 (Fig. 1*C*). *h*_ℓ_(**x_s_ − *x*_*o*_**) is the convolution of a Gaussian spatial profile with standard deviation $\sigma_{in}=0.09^{\circ }$ with the square stimulus taken to have height 1. It is given by

$$\begin{array}{rl} h_{\ell }(x_{s}-x_{o}) & =\frac{1}{4}\left(\text{erf}\left(\frac{\ell /2+(x_{s}-x_{o})}{\sigma_{in}\sqrt{2}}\right)+\text{erf}\left(\frac{\ell /2-(x_{s}-x_{o})}{\sigma_{in}\sqrt{2}}\right)\right)\\ & \;*\;\left(\text{erf}\left(\frac{\ell /2+(y_{s}-y_{o})}{\sigma_{in}\sqrt{2}}\right)+\text{erf}\left(\frac{\ell /2-(y_{s}-y_{o})}{\sigma_{in}\sqrt{2}}\right)\right), \end{array}$$

where erf(x) is the error function defined as $\text{erf}(x)=\frac{1}{\sqrt{\pi }}\int_{-x}^{x}e^{-t^{2}}dt$. The function *g* is defined by

$$g(|\theta_{s}-\theta_{o}|)=e^{\frac{-|\theta_{s}-\theta_{o}{|}^{2}}{2\;*\;\sigma_{\;fori}^{\;2}}}$$

with $\sigma_{\;fori}=20^{\circ }$.

For brevity we use superscript letters *a*, *b*, … rather than function argument (**x_a_**), (**x_b_**), …. To define the rate equations, we let $r_{E}^{a}$ be the rate of the excitatory neuron at position **x_a_**, and $r_{I}^{a}$ similarly. Both receive the same external input $I_{ext}^{a}$ given by $\Sigma_{C\theta_{s}}(x_{o})$ of Equation 2, with the neuron’s location now specified by *a* rather than *x*_*o*_. The rate equations are

$$\begin{array}{cc} & \tau_{E}\frac{dr_{E}^{a}}{dt}=-r_{E}^{a}+K[I_{ext}^{a}+\sum_{b}W_{EE}^{ab}r_{E}^{b}-\sum_{b}W_{EI}^{ab}r_{I}^{b}{]}_{+}^{n_{E}},\\ & \tau_{I}\frac{dr_{I}^{a}}{dt}=-r_{I}^{a}+K[I_{ext}^{a}+\sum_{b}W_{IE}^{ab}r_{E}^{b}-\sum_{b}W_{II}^{ab}r_{I}^{b}{]}_{+}^{n_{I}}, \end{array}$$

where $\left[x{]}_{+}=\max(0,x\right)$. The excitatory cells’ time constant *τ*_*E*_ = 10 ms, and the inhibitory cells’ time constant *τ*_*I*_ = 6.67 ms. We use, *K* = 0.01 a.u., *n*_*E*_ = *n*_*I*_ = 2.2. $\sum_{b}W_{XE}^{ab}r_{E}^{b}$ is the recurrent excitatory input to neuron *X*^*a*^ where *X* = {*E*, *I*}. Similarly, $\sum_{b}W_{XI}^{ab}r_{I}^{b}$ is the recurrent inhibitory input. The inputs are in arbitrary units (a.u.) because the form of Equation 6 allows us to absorb any units in the definition of *K*.

For the conductance-based model, we ran simulations using the Brian simulator (Stimberg et al., 2019). The equations of motion of the membrane potential and the conductances for each cell are identical for E and I cells. For a cell at *a* of type *X*, the equations are (we omit specifying the type *X* for the dynamical variables and parameters that don’t differ between the two types):

$$\begin{array}{rl} & \tau_{m}\frac{dV^{a}}{dt}=-(V^{a}-R_{L})+\frac{g_{E}^{a}}{g_{L}}(R_{E}-V^{a})+\frac{g_{I}^{a}}{g_{L}}(R_{I}-V^{a})+\frac{g_{in}^{a}}{g_{L}}(R_{E}-V^{a})+\sigma_{\;V}\sqrt{2\tau_{m}}\eta^{a}(t),\\ & \tau_{E}\frac{dg_{E}^{a}}{dt}=-g_{E}^{a}+\tau_{E}\sum_{b=1}^{N_{E}}\sum_{j}g_{XE}^{ab}\delta (t-t_{Ej}^{b}),\\ & \tau_{I}\frac{dg_{I}^{a}}{dt}=-g_{I}^{a}+\tau_{I}\sum_{b=1}^{N_{I}}\sum_{j}g_{XI}^{ab}\delta (t-t_{Ij}^{b}),\\ & \tau_{E}\frac{dg_{in}^{a}}{dt}=-g_{in}^{a}+{\bar{g}}_{in}^{a}+\sqrt{\tau_{E}}\sigma_{g_{in}}^{\;a}\zeta^{\;a}(t). \end{array}$$

Here, *V*^*a*^ is the membrane potential of the given cell at a. *τ*_*m*_ is the membrane potential time constant. $g_{E}^{a}$ is the excitatory AMPA-like conductance, $g_{I}^{a}$ is the inhibitory GABA-like conductance and $g_{in}^{a}$ is the excitatory input conductance from outside the network. *R*_*E*_, *R*_*I*_, and *R*_*L*_ are reversal potentials of the excitatory, inhibitory, and leak conductances. *τ*_*E*_ and *τ*_*I*_ are the time constants of the excitatory and inhibitory conductances. $g_{XE}^{ab}$ is the conductance of the synapse of the excitatory cell at *b* to the given cell at *a*, similarly $g_{XI}^{ab}$ is the conductance from the inhibitory cell at *b*, and $t_{Xj}^{b}$ is the time of the *j*th spike of the cell of type *X* at *b*. *δ*(*x*) is the Dirac delta function. Each cell in the network receives input from *N*_*input*_ external spiking cells, where *N*_*input*_ is a large number. We assume the spike trains are Poisson and invoke the central limit theorem to approximate the input to the cell $\tau_{E}g_{ext}\sum_{i=1}^{N_{input}}\sum_{k}\delta (t-t_{i}^{k})$ by a stochastic process with mean ${\bar{g}}_{in}^{a}$ and variance $\tau_{E}\sigma_{g_{in}}^{a^{2}}$, where *ζ* ^*a*^ is white Gaussian noise with <*ζ* ^*a*^(*t*)*ζ* ^*a*^(*t*′) > =*δ*(*t* − *t*′). The stochastic dynamics will lead $g_{in}^{a}$ to have a mean ${\bar{g}}_{in}^{a}$ and a variance $\sigma_{g_{in}}^{a^{2}}/2$ (Tuckwell, 1988a), where

$$\begin{array}{cc} & {\bar{g}}_{in}^{a}=N_{input}r_{ext}^{a}\tau_{E}g_{ext},\\ & \sigma_{g_{in}}^{\;a^{2}}=N_{input}r_{ext}^{a}\tau_{E}g_{ext}^{2}. \end{array}$$

where *g*_*ext*_ is the amplitude of the conductance evoked when a single external cell spikes, and $r_{ext}^{a}$ is the firing rate of the external cells given by Equation 2. We assume the membrane potential is noisy, and take the noise to be white Gaussian noise. The membrane fluctuations are described by an Ornstein-Uhlenbeck (OU) process (Uhlenbeck and Ornstein, 1930; HC Tuckwell, 1988b). *η*^*a*^(*t*) is a Gaussian random variable with mean 0 and variance 1, and *σ*_*V*_ is the standard deviation of the membrane potential fluctuations. In the simulations we set *σ*_*V*_ = 6.85 mV to get spontaneous activity similar to what has been reported in Chen et al. (2009), Ringach et al. (2002), and Gur and Snodderly (2008). In the model the mean spontaneous activity of the excitatory cells is about 1.5 Hz, and of the inhibitory cells is about 3 Hz. The parameters for both E and I cells are as follows: *τ*_*m*_ = 15 ms; *τ*_*E*_ = *τ*_*I*_ = 3 ms; *R*_*L*_ = −70 mV; *R*_*E*_ = 0 mV; *R*_*I*_ = −80 mV; *g*_*L*_ = 10 nS; *N*_*input*_ = 200 and *g*_*ext*_ = 0.1 nS. We take threshold voltage *V*_*th*_ = −50 mV and after-spike rest voltage to be 6 mV below threshold, *V*_*r*_ = −56 mV, as in Troyer and Miller (1997). After the cell spikes, it goes into a refractory period with *τ*_*ref*_ = 3 ms. Similar to the rate model, the conductance values are given by $g_{XY}^{ab}=g_{XY}p_{XY}(|x_{a}-x_{b}|)q_{XY}(|\theta_{a},\theta_{b}|)$, where the functions *p*_*XY*_ and *q*_*XY*_ are as defined previously. The parameters *g*_*XY*_ are: *g*_*EI*_ = 3.3 nS and *g*_*II*_ = 2 nS; at distances less than *L*_*o*_, *g*_*EE*_ = 1.8 nS and *g*_*IE*_ = 1.76 nS; and at distances greater than *L*_*o*_, *g*_*EE*_ = 0.7 nS and *g*_*IE*_ = 0.65 nS. Again *L*_*o*_ = 324 μm as in the rate model.

For the conductance-based model, parameters such as the membrane time constant, time constants for the excitatory and inhibitory conductances, reversal potentials, threshold voltage, and refractory period were chosen to be within biological ranges and are values widely used in models (e.g., see multiple examples of models at the Brian simulator (Stimberg et al., 2019) website, https://brian2.readthedocs.io/en/stable/examples/index.html). Other parameters, such as the noise standard deviation or conductance values, were chosen so that the spontaneous activities, E and I cell firing rates, and measured conductance values are in line with experimental data. We checked initial simulations to ensure that the network was operating in the asynchronous, irregular regime; e.g., C.V.’s of interspike intervals for both E and I cells during visual stimulation were generally in the range 0.6–1.2, with the bulk in the range 0.7–1.

We have studied both the rate model and the conductance-based model under small perturbations of most of the parameters and observed no obvious changes in behaviors. We also more extensively studied variations of different parameters to explore their effect on behavior, particularly in the rate model. Some parameter combinations lead to instability, but otherwise the findings were quite robust. We chose the model parameters through the various considerations described above. Moreover, we endeavor to understand and explain the mechanisms underlying our results, which do not depend on fine details. For all of these reasons, we believe the results are robust and have no need of fine tuning of parameters.

To measure the strength of the surround suppression in the network, we compute the suppression index (SI) defined as follows:

$$SI=\frac{r_{\max}-r_{inf}}{r_{\max}},$$

where $r_{\max}$ is the response to a stimulus size that elicits maximum response, and *r*_*inf*_ is the response for a very large stimulus. To measure whether the presence of a surround stimulus facilitates or suppresses the response of a cell compared to its response to a center-only stimulus, we define a modified suppression index:

$$SI_{m}=\frac{r_{\left(center-only\right)}-r_{\left(center+surround\right)}}{r_{\left(center-only\right)}},$$

where *SI*_*m*_ negative means facilitation, while *SI*_*m*_ positive means suppression.

To describe our square shaped stimuli, we use the words width or stimulus size to refer to the length of the sides of the square, and radius to refer to half of the width. We describe an annulus by the width of squares constituting its inner and outer borders. In experiments on the surround tuning to the center orientation, we fix the center stimulus width and the inner and outer annulus widths to $(1.3^{\circ },4.3^{\circ }$, and $21.6^{\circ })$ respectively, and set both stimulus contrasts to 100. In these experiments, we record the activity of a single neuron as we vary the stimulus orientations, and we roughly pick the largest annulus inner width at which the phenomena is still observed. This corresponds to an annulus inner radius of $2.15^{\circ }$ or 1.1 mm, which is roughly the span of E-to-I monosynaptic connections in the model. In feature-specific suppression experiments, we fix the center stimulus width and the inner and outer annulus widths to $(1.7^{\circ },3.9^{\circ }$, and $21.6^{\circ })$, respectively. In these experiments we follow the procedure in Trott and Born (2015) to make our results directly comparable with experimental data. Thus, we use a slightly bigger center stimulus to obtain a better fit of the population rates (see Result 3 for more details). The contrast is set to 16.4 (representing 80% of the maximal external input strength, *C* = 100), for each component of the plaid as well as the surround in the rate model, and to 50 (representing 99.5% of the maximal external input strength, *C* = 100) in the conductance-based model.

In all experiments, cells are sampled from locations away from the boundary. We first randomly pick 100 locations within the region we define as away from the boundary (20 < *x* < 60, 20 < *y* < 60). Experiments are then performed on a set of cells randomly picked from those 100 locations (independently sampled for each figure unless otherwise stated).

## Code Accessibility

The code/software described in the paper is freely available online at https://github.com/DiObeid/SSN_2d_V1.

10.1523/ENEURO.0459-24.2025.d1 Extended data Download Extended data, ZIP file.

## Results

**Figure 3.**
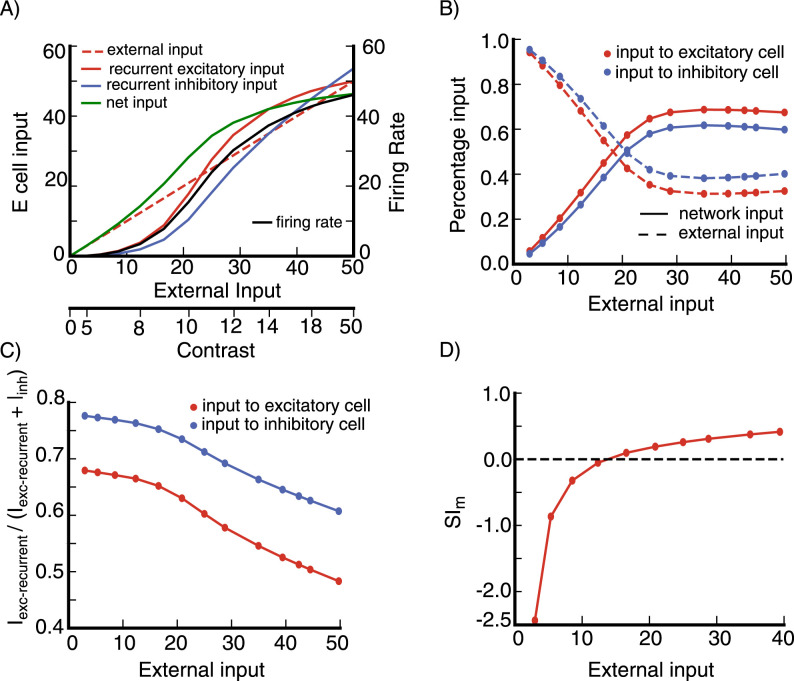
SSN behavior of the model network ***A***, Inputs to an excitatory (***E***) cell and its firing rate vs external input. The cell is at a randomly selected grid location (see Section Model, subsection Model details). Stimulus contrast level corresponding to external input is shown on the bottom axis. The net input is defined as (*I*_*ext*_ + *I*_*exc*−*recurrent*_ − *I*_*inh*_), where *I*_*ext*_ is the external input to the cell, *I*_*exc*−*recurrent*_ is the cell’s recurrent excitatory input from the network, and *I*_*inh*_ its recurrent inhibitory input from the network, *I*_*inh*_ is defined to be positive, see Section Model, subsection Model details for the expressions of *I*_*exc*−*recurrent*_ and *I*_*inh*_. ***B***, The network is dominated by external input for weaker stimuli (weaker external input) and by network (recurrent) inputs for stronger stimuli. Plot shows percentage of external and network inputs as a function of external input for the excitatory cell in (***A***) and an inhibitory cell at the same grid location (dashed line is external input, solid line is network input). Here, the total input is defined as (*I*_*ext*_ + *I*_*exc*−*recurrent*_ + *I*_*inh*_), and the network input is (*I*_*exc*−*recurrent*_ + *I*_*inh*_). ***C***, Network input is increasingly inhibition-dominated with increasing stimulus strength. Plot shows percentage of network input that is excitatory, *I*_*exc*−*recurrent*_/(*I*_*exc*−*recurrent*_ + *I*_*inh*_), as a function of external input for the excitatory and inhibitory cells in (***A***, ***B***). In panels (***A***–***C***) we use a stimulus of width 2.16° centered on the cell’s retinotopic position and with the cell’s preferred orientation. ***D***, Surround Facilitation to Suppression transition: a near surround can be facilitating or suppressing depending on the center stimulus contrast. *SI*_*m*_ negative means facilitation, while *SI*_*m*_ positive means suppression (see Section Model, subsection Model details, Eq. 10 for the definition of *SI*_*m*_). In panel (D), the data are from an excitatory cell at a randomly selected grid location (see Section Model, subsection Model details); surround stimulus has contrast *C* = 12, and inner and outer widths 0.865° and 4.32° respectively; the center stimulus width is 0.65° and its contrast is shown on the x-axis; both center and surround stimuli are centered on the cell’s retinotopic position, with the cell’s preferred orientation. The inputs are in arbitrary units (a.u.).

We first check that our network is functioning as an SSN, by checking for several salient SSN behaviors. The SSN shows a transition, with increasing input strength, from a weakly coupled, largely feedforward driven regime for weak external input, to a strongly coupled, recurrently-dominated regime for stronger external input (Ahmadian et al., 2013). This transition can account for many aspects of summation of responses to two stimuli and of center-surround interactions and their dependencies on stimulus contrast (Rubin et al., 2015). Our network shows the characteristic signs of this transition (Rubin et al., 2015): the net input a neuron receives grows linearly or supralinearly as a function of external input for weak external input, but sublinearly for stronger external input (Fig. 3*A*); this net input is dominantly external input for weak external input, but network-driven input for stronger external input (Fig. 3*B*); the network input becomes increasingly inhibitory with increasing external drive (Fig. 3*C*); and a surround stimulus of a fixed contrast can be facilitating for a weak center stimulus, but becomes suppressive for stronger external drive to the center (Fig. 3*D*).

We then explore whether lateral connections in V1 are capable of generating several phenomena that emerge due to center-surround interaction.

### Surround suppression

We first investigate surround suppression, a widely studied phenomena in V1 and other sensory areas in multiple species (Angelucci et al., 2017). To study surround suppression, we record the firing rates of a cell in the network, as we vary the width of a high contrast stimulus centered on the cell’s retinotopic position and with orientation identical to the recorded cell’s preferred orientation.

**Figure 4.**
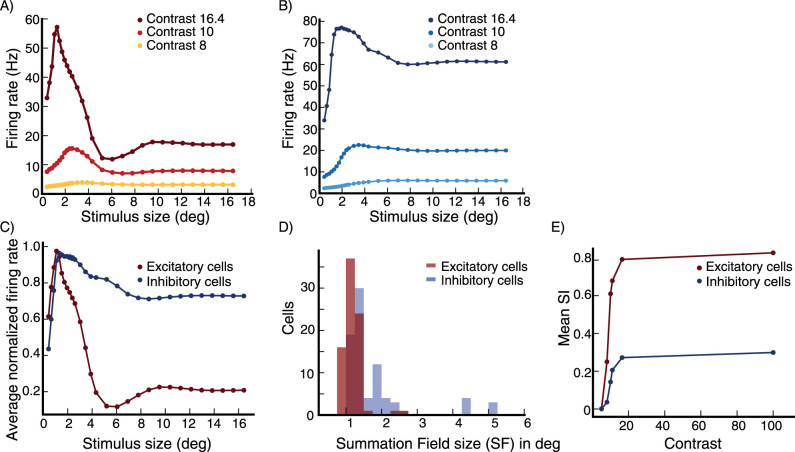
Surround suppression (***A***, ***B***) The firing rates of an excitatory (***E***) cell (***A***) and an inhibitory (***I***) cell at the same grid location (***B***) vs. stimulus size. Different colors correspond to different stimulus contrast levels, high (*C* = 16.4; external input 80% of maximal), medium (*C* = 10; external input 42% of maximal) and low (*C* = 8; external input 25% of maximal). The cells are at a randomly selected grid location (see Section Model, subsection Model details). ***C***, The average firing rate of 80 E cells at randomly selected grid locations (see Section Model, subsection Model details), and of 80 I cells at the same grid locations, after normalizing each cell’s rates so that its peak rate is 1.0, vs. stimulus size for a high contrast stimulus (*C* = 16.4). ***D***, The distribution of Summation Field sizes (SFS) of the E and I cells used to produce panel (***C***), the mean SFS for the E cells is 1.14 deg and for the I cells is 1.75 deg. ***E***, The mean suppression index of the E cells and I cells used to produce panel (***C***), versus stimulus contrast. The mean Suppression Index (SI) for E and I cells changes from little or no suppression (low SI’s) for very weak stimuli to stronger suppression (higher SI’s) for stronger stimuli, with E cells showing much stronger suppression than I cells. The error bars are of order 10^−2^ or smaller.

We first show that our model replicates surround suppression behavior and its contrast dependence. In the model, both excitatory (E) and inhibitory (I) cells are surround-suppressed. However, excitatory cells are more strongly surround suppressed than inhibitory cells, as illustrated by an E and I cell at a randomly selected grid location (see Section Model, subsection Model details) (Fig. 4*A,B*) and by the average size tuning across 80 E and 80 I cells (Fig. 4*C*) for a high contrast stimulus, *C* = 16.4. Accordingly, the summation field sizes—the size of a stimulus driving optimal response, before further increase in size causes response suppression—of E cells are smaller than those for I cells (Fig. 4*D*).

**Figure 5.**
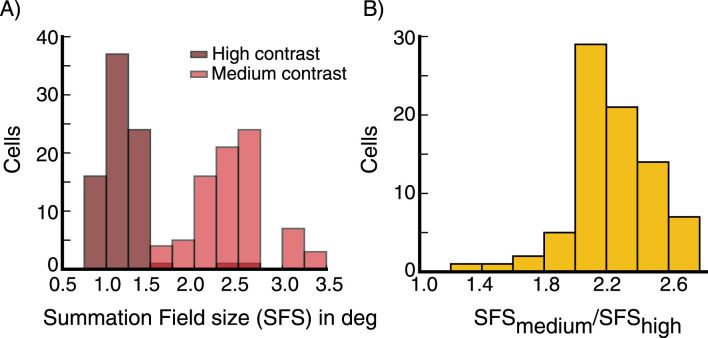
Surround suppression, summation field sizes ***A***, The distribution of summation field sizes for 80 excitatory (***E***) cells (same E cells used to produce panel Fig. 4*C*), at contrast *C* = 16.4 (dark red color) and contrast *C* = 10 (light red color). ***B***, The distribution of the ratio of the summation field sizes in (***A***). The summation field size of all cells is smaller for the higher contrast stimulus.

We repeat the above experiment with different contrast levels. The strength of surround suppression increases with increasing stimulus contrast (Fig. 4*A*,*B*). The mean suppression index (SI) increases from little or no suppression for weak contrasts to stronger suppression for stronger contrasts (Fig. 4*E*). For a relatively high contrast stimulus (*C* = 16.4, representing 80% of the maximal input strength), the mean suppression index (SI) is 0.79 for the E cells and 0.27 for the I cells (where 0 is no suppression and 1 is complete suppression). Similarly, the summation field size shrinks with increasing contrast, as we illustrate for E cells in Figure 5*A*,*B*, as in Sceniak et al. (1999). The summation field sizes of I cells behave similarly.

**Figure 6.**
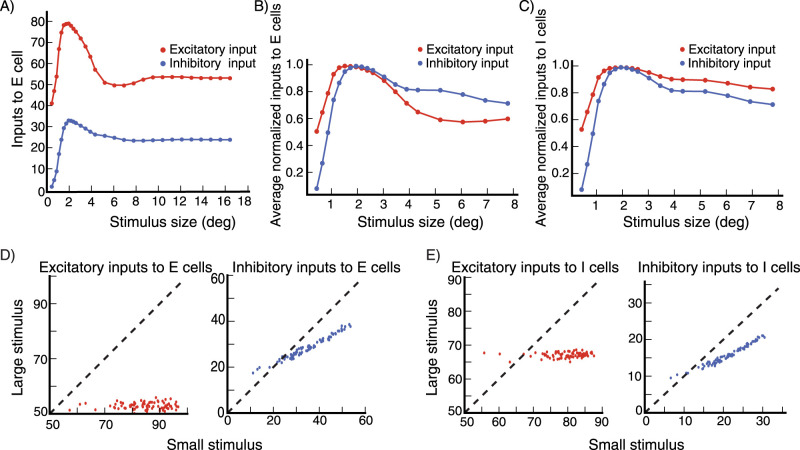
Surround suppression, inputs to cells ***A***, The excitatory (red) and inhibitory (blue) total input to the excitatory (***E***) cell in Figure 4*A*, shown for the high contrast stimulus (*C* = 16.4; external input 80% of maximal), both show surround suppression. (***B***, ***C***) The size tuning of the averaged normalized excitatory and inhibitory inputs (each normalized to have peak value 1) to excitatory (***E***) cells (***B***) and inhibitory (***I***) cells (***C***) for contrast *C* = 16.4 (same cells used to produce panel Fig. 4*C*). Note the change in horizontal axis between panels (***A***) and (***B***, ***C***). ***D***, The excitatory and inhibitory inputs to E cells (same E cells used to produce panel Fig. 4*C*) for a large stimulus (for which all the cells are surround suppressed) are shown vs. their values for a small stimulus (with size given by the average size that yields maximal response across cells, see Section Results, subsection Surround suppression). Panel (***E***) is the same as (***D***) but for I cells (same I cells used to produce panel Fig. 4*C*). Stimulus contrast *C* = 16.4. The inputs are in arbitrary units (a.u.).

We next examine whether surround suppression in the network is accompanied by a decrease in inhibition as well as excitation received by cells (see Section Introduction), as reported by Ozeki et al. (2009) and Adesnik (2017), rather than simply being due to ramping up of inhibitory input. The size tuning of the excitatory and inhibitory input currents to the E cell in Figure 4*A* at high contrast (*C* = 16.4) reveals that both currents indeed show surround suppression (Fig. 6*A*). We then look at the average size tuning of these currents across cells, after normalizing each cell’s curve for each current to have a peak of 1. Both E cells (Fig. 6*B*) and I cells (Fig. 6*C*) show surround suppression of both their excitatory and their inhibitory currents.

We then wish to directly compare, across cells, the currents for a small, nearly optimally sized stimulus to those for a large, suppressive stimulus. To compare to experiments, we must consider that experimenters cannot know the exact optimal size, and must choose some size in that vicinity, which may evoke less inhibition than the peak (see Fig. 4). Thus, if in our model we choose the optimal size for comparison to the surround-suppressed state, we may bias our results towards seeing a decrease in inhibition, compared to experimental procedures. To avoid this, we follow a procedure similar to that of Ozeki et al. (2009). We measure the excitatory and inhibitory inputs for a small stimulus size with width *d*_*s*_ around which the cells respond close to maximally, and for a very large stimulus at which all cells are surround suppressed. We take *d*_*s*_ to be equal to the median of all stimulus widths for which the sampled cells respond maximally. The results are entirely similar if *d*_*s*_ is taken to be the mean rather than the median. Using this procedure, for excitatory inputs and for inhibitory inputs to E and to I cells, we plot the input current at small stimulus size vs. the current at large stimulus size (Fig. 6*D*,*E*). In Figure 6*D* we plot the excitatory inputs and inhibitory inputs respectively to 80 excitatory cells, at small stimulus size against those at large stimulus size. Both excitatory and inhibitory inputs are smaller for the large suppressive stimulus. Figure 6*E* shows the same data for 80 inhibitory cells. Thus, for both excitatory and inhibitory cells in the model, surround suppression is accompanied by a decrease in excitation and inhibition that the cell receives.

What do these results depend on? While we cannot exhaustively search all parameters, in our explorations of parameters, we have found that surround suppression of inhibitory as well as excitatory input depends on two elements of the connectivity. First, locally, roughly over distances of about *L*_*o*_ (the distance over which lateral connections are most dense, see section Model Details), the cells must be strongly enough connected so that, as the stimulus size increases, the local circuit around the recorded cell goes through the SSN transition from being mainly driven by the feedforward input to being dominated by recurrent currents. This occurs through the increase in effective synaptic weights with increased external drive to the network due to the expansive, supralinear neuronal input/output function, which is the fundamental mechanism underlying the SSN (see Ahmadian et al., 2013; Rubin et al., 2015 for a detailed description of the SSN mechanism). At the transition, the growth of effective excitatory synaptic strengths is sufficient that the excitatory subnetwork becomes unstable by itself (Ahmadian et al., 2013), but the network is stabilized by feedback inhibition. This means that the local circuit becomes an inhibition stabilized network (ISN), which was the condition for surround suppression of inhibitory input identified in Ozeki et al. (2009), based on the ISN mechanism initially identified by Tsodyks et al. (1997). We note that our network is in a regime of the SSN that is thought to show the most strongly sublinear response summation (see Section Model, subsection Model details).

The second element we have found critical is that the ratio of projection strength of long-range horizontal connections to I cells vs. to E cells must increase with increasing distance, that is, the E-to-I connections must be effectively longer range than E-to-E connections. Furthermore, the excitatory input received by I cells from far away E cells should not be large compared to the excitatory input they receive from nearby excitatory cells. Then, with increasing stimulus size, the loss of excitatory input to I cells from surround suppression of nearby E cells can exceed the gain of excitatory input from far away E cells, causing the I cells to be surround suppressed. Note that, in our model (as in Rubin et al., 2015), the I cells have larger summation fields than the E cells (Fig. 4*C*,*D*). This means that there is an intermediate range of stimulus sizes for which inhibitory firing rates continue on average to increase with stimulus size, while excitatory cells are surround suppressed. With further increase in stimulus size, both E and I cells are suppressed.

### Surround tuning to the center orientation

Cells in V1 are suppressed maximally when the surround stimulus orientation matches the center stimulus orientation, regardless of whether that orientation matches the cell’s preferred orientation (Sillito et al., 1995; Shen et al., 2007; Shushruth et al., 2012; Trott and Born, 2015).

The previous model (Shushruth et al., 2012) showed that this behavior could arise if the local network’s connectivity was broadly tuned for orientation and strong, while the input from the surround was narrowly tuned. Then, when a non-preferred orientation is presented in a cell’s receptive field, its strongest input would come from the most active local cells, those with preferred orientation matching the center stimulus orientation. The cell would then be most suppressed by surround input that targets and most strongly suppresses those most active cells, which are its main source of input. That is, the cell would be most suppressed when the surround orientation matches the center orientation.

We use similar reasoning here, but now in the context of the SSN model with power-law rather than linear-rectified input/output functions. Our long-range projections are excitatory onto both E and I cells, whereas in Shushruth et al. (2012) they were inhibitory onto E cells and excitatory onto I cells. In addition, the model of Shushruth et al. (2012) was not recurrent, because the input from one cell to another was simply determined by the difference in their preferred orientations, regardless of the firing rate of the presynaptic cell (and thus was a constant, independent of the stimulus); and the surround input to a cell was determined only by the difference between the cell’s preferred orientation and the surround stimulus orientation, and not by the firing rates of lateral cells responding to the surround stimulus. In contrast, our model is a recurrent model.

**Figure 7.**
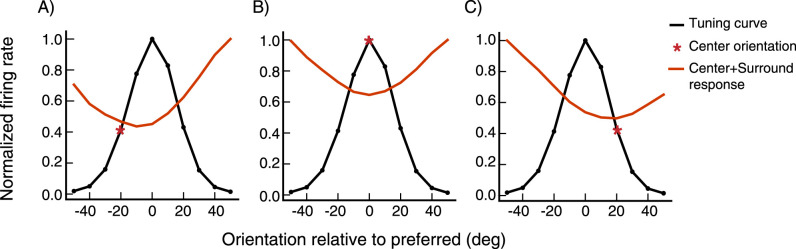
Surround tuning to the center orientation in an excitatory (***E***) cell The orientations are relative to the cell’s preferred orientation. The black curves show the cell’s orientation tuning curve for a center-only stimulus (i.e., firing rate vs. center orientation), normalized so the maximum response is 1.0. The red curves show the similarly normalized tuning to surround orientation for a fixed center stimulus. In each panel, the red asterisk marks the fixed center orientation: ***A***, center at preferred minus 20°, (***B***) center at preferred and (***C***) center at preferred plus 20°. This cell is at a randomly sampled grid location (see Section Model, subsection Model details).

**Figure 8.**
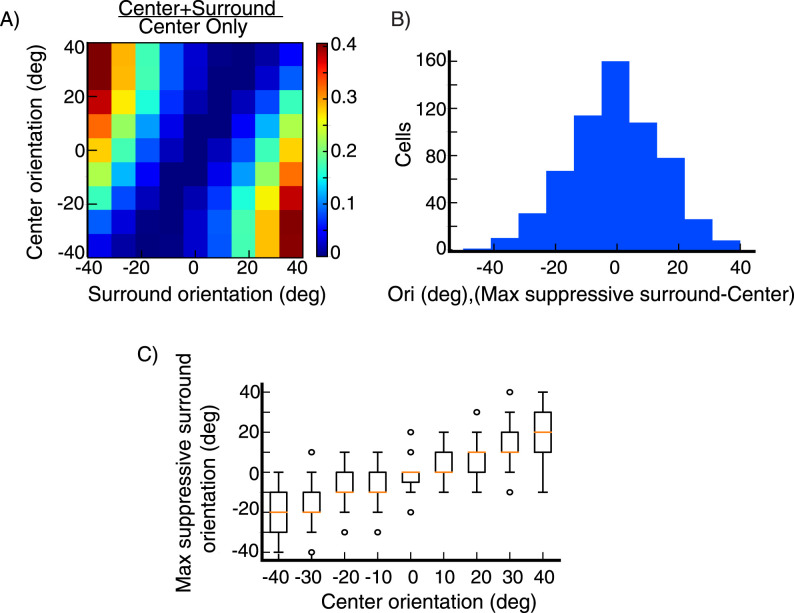
Surround tuning to the center orientation Surround tuning in 67 excitatory (***E***) cells at randomly selected grid locations (see Section Model, subsection Model details). Both center and surround orientations are varied from preferred minus 40° to preferred plus 40° in increments of 10°. ***A***, Average surround modulation map. For each cell, the map is obtained by dividing center-surround responses by the corresponding center-only responses, each row has its minimum value subtracted. ***B***, Histogram of the difference between the orientation of the surround that maximally suppresses the cell’s firing rate, and the center stimulus orientation. The data is pooled over all cells and center orientations. ***C***, Whisker plot of orientation of the surround that maximally suppresses the cell’s firing rate against the center stimulus orientation, the box extends from quantile Q1 to Q3, the orange line is the median. The upper whisker extends to last datum less than Q3 + k * IQR, similarly, the lower whisker extends to the first datum greater than Q1 − k * IQR, where IQR is the interquartile range (Q3-Q1) and *k* = 1.5, the circles represent the outlier data. In (***A***) and (***C***) orientations are shown relative to preferred.

We record the firing rate of cells in the network for different center orientations. For each center orientation, we then present a stimulus in the surround, rotate its orientation, and record the cell’s firing rate for each center-surround orientation configuration. In an excitatory cell (Fig. 7), we study tuning to center orientation absent a surround (black curves) and then tuning to surround orientation for a fixed center stimulus (red curves). The most suppressive surround orientation (minimum of red curve) is pulled strongly toward the center orientation (red asterisk) as the center orientation is varied from $-20^{\circ }$ relative to preferred orientation (Fig. 7*A*), to preferred (Fig. 7*B*), to +20° relative to preferred (Fig. 7*C*). Similar results are seen more generally in 67 excitatory cells at randomly selected grid locations (Fig. 8). The surround orientation producing maximum suppression is pulled strongly towards the center orientation (Fig. 8*A*,*C*) and in most cases is within 10° of the center orientation (Fig. 8*B*).

As described above, surround tuning to the center orientation arises due to the strong, broadly tuned local connectivity profile in orientation space, along with the more sharply tuned surround input, which causes maximal input to the cell to come from cells preferring the center stimulus rather than from cells with the same preferred orientation as the recorded cell. This makes suppression targeting cells that prefer the center stimulus orientation more potent than suppression targeting cells that prefer the same orientation as the recorded cell.

### Feature-specific surround suppression

Surround suppression in V1 is not blind to the center stimulus, as we have just seen. Trott and Born (2015) argued that this could be compatible with two scenarios. In what they call an output-gain model, surround suppression would be a form of “normalization” of the local circuit that roughly equally suppresses all cells, regardless of their preferred orientation or more generally of the relationship between their feature preferences and the stimuli. This suppression would be strongest when center and surround orientations match, but it would apply equally to all cells. In what they call an input-gain model, surround suppression specifically suppresses the component of a neuron’s input that matches the surround stimulus. In this case, the degree of suppression could vary with a cell’s stimulus preferences. In seeming support of the output-gain model, they found that, binning cells by their preferred orientation, the average degree of suppression (ratio of center-plus-surround response to center-only response) to a given center-surround stimulus combination was the same for cells of all preferred orientations. However, this could also be compatible with an input-gain model, if cells received input from all orientations according to an input tuning curve centered on the cell’s preferred orientation.

To distinguish these scenarios, they studied *feature-specific* surround suppression. They showed two simultaneous drifting gratings—a plaid stimulus—in the cell’s center, and showed a surround stimulus with orientation corresponding to one of the two gratings. They found that the response component driven by the center grating whose orientation matches the surround’s was most suppressed, which they took as evidence for the input-gain model. We tested whether our model would also show this property, following the procedure described in Trott and Born (2015).

**Figure 9.**
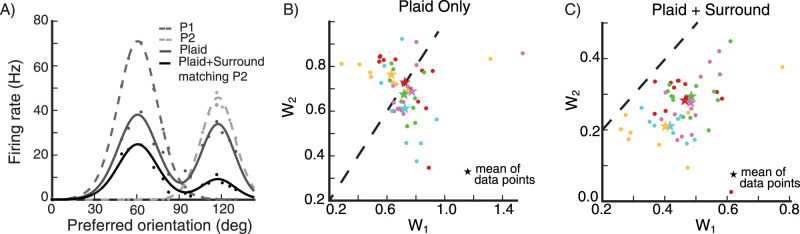
Feature-specific surround suppression ***A***, The firing rate of a small population of neurons in response to a center stimulus. The neurons are binned in 5° bins according to their preferred orientation. The dots are the data points, and the lines are the von-Mises-function fits to the data. The medium gray points and dashed line are the population response to the first component of the plaid (P_1_). The light gray points and dashed line are the population response to the second component of the plaid (P_2_). The dark gray points and solid line are the population response to the plaid. The black points and solid line are the population response to the plaid in the presence of a surround stimulus whose orientation matches the plaid’s second component. ***B***, ***C***, values of w_1_ and w_2_ (the weightings in fitting the plaid population response to a weighted sum of the two component responses), for a 60° center plaid stimulus, shown for 12 different plaid rotations (every 10°), recorded from five different populations (indicated by colors). The populations are centered around randomly selected grid locations (see Section Model, subsection Model details). Missing data points imply that there is no good fit of the data for certain stimulus configurations. The star symbols are the mean values of w_1_ and w_2_ for each population. ***B***, Responses to plaid center stimulus only. ***C***, Responses to plaid center stimulus in the presence of a surround stimulus with orientation equal to the plaid’s second component. Dashed lines are unit diagonals, along which w_1_=w_2_.

We record the firing rate of a small population of neurons to each of two oriented gratings, that if shown superimposed would form a plaid. For each stimulus, we fit the average response vs. preferred orientation across the population with a von Mises function, call these functions P_1_ and P_2_ (Fig. 9*A*). We then record the population’s firing rates to a center plaid stimulus, the superposition of the two individual gratings. If the two gratings differ by, e.g., $60^{\circ }$, we will call this a $60^{\circ }$ plaid or a plaid angle of $60^{\circ }$. We fit the population’s response to the plaid stimulus as a linear combination of the two components, R_plaid_ = w_1_ P_1_ + w_2_ P_2_. Afterwards, we introduce a surround stimulus whose orientation matches the second component of the plaid center stimulus, and find the new values of w_1_ and w_2_. We then repeatedly rotate the plaid stimulus in increments of 10 degrees, and find new values of w_1_ and w_2_ for each orientation of the plaid, each time matching the surround stimulus orientation to the orientation of the second plaid component.

For responses to a 60° plaid alone, w_1_ and w_2_ on average have about equal strength, but the addition of a surround stimulus matched to the second plaid component suppresses w_2_ much more than w_1_ (Fig. 9*B*,*C*).

**Figure 10.**
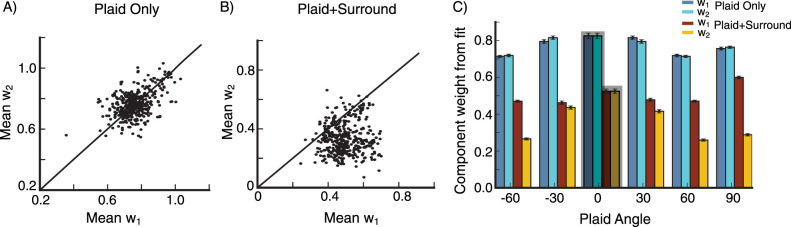
Feature-specific surround suppression ***A***, ***B***, Mean w_1_ is plotted against mean w_2_ for plaid angles $\left[-\;60^{\circ },-\;30^{\circ },30^{\circ },60^{\circ },90^{\circ }\right]$ from 80 populations, centered around 80 randomly selected grid locations (see Section Model, subsection Model details). The mean values of w_1_ and w_2_ are obtained from averaging data for different rotations of the plaid (***A***) for plaid center stimulus only (***B***) for plaid center stimulus in the presence of surround stimulus with orientation equal the plaid’s second component. ***C***, Mean values of the data points in (***A***) and (***B***) for different plaid angles, we also include the data for plaid angle 0°, error bars are the s.e.m.

We carry out this experiment for different plaids, with plaid angles [$-60^{\circ }$, $-30^{\circ }$, 0°, 30°, 60°, 90°]. The mean values of w_1_ and w_2_ across all of these plaids cluster around w_1_=w_2_ for the plaid stimulus alone (Fig. 10*A*), but are heavily shifted towards w_1_ when the surround stimulus matched to the second plaid component is added (Fig. 10*B*). In Figure 10*C* we show the mean values of data points in Figure 10*A*,*B* for each plaid angle. In the absence of a surround stimulus there is no difference between w_1_ and w_2_. When a surround stimulus with orientation matching the plaid’s second component is introduced, both components of the plaid are suppressed, however, we clearly see that the second component is suppressed more. Hence, the surround stimulus most strongly suppresses the center stimulus component of the same orientation as the surround. (The results remain the same if we repeat the same experiment and record from single cells rather than from a small local population, not shown).

Thus, our model results, like the experimental results of Trott and Born (2015), are consistent with what they call the input-gain model: specific suppression of the component of a neuron’s input that matches the surround stimulus. The mechanism of this “input-gain” is simply that the long-range projections connect cells in surround and center of similar preferred orientation, so that the surround of a given orientation specifically suppresses the component of response due to the same orientation in the center. We found (not shown) that this mechanism gives the same result whether or not the circuit includes the strong and broadly-orientation-tuned local connectivity that was required to explain surround tuning to the center orientation (i.e., the strongest suppression arising when surround orientation matches center orientation, even when the center orientation is not the cell’s preferred orientation). That is, the mechanism underlying the input-gain model is not sufficient to explain surround tuning to the center orientation; the latter requires, in addition, local connectivity that leads a cortical cell to receive its strongest input to a non-preferred stimulus orientation from cortical cells preferring the stimulus orientation.

### Activity decay time

We now study the intrinsic time constant of our model circuit, by examining the time course of activity decay when external input to the circuit is removed. We are motivated by results showing that, in the mouse, both in V1 (Reinhold et al., 2015) and in a pre-motor cortical area, area ALM (Guo et al., 2017), silencing of the thalamic input to the area caused activity to decay away with a roughly 10 ms time constant, comparable to the time constants of individual neurons (Schoonover et al., 2014). This result was surprising, for the following reasons. The recurrent excitatory-to-excitatory connectivity seen in cortex can create long network activity timescales (technically, if there are eigenvalues with positive real part in the linearization of the model’s connectivity matrix about the model’s fixed points, e.g., Murphy and Miller, 2009), because activity decay is partially countered by recurrent excitatory input, slowing the decay. Cortical areas have activity decay times (as measured by autocorrelation times) much longer than 10 ms under normal conditions, ranging in primate from roughly 65 ms in primary sensory areas to 200–350 ms in motor and frontal areas (Murray et al., 2014), and a similar hierarchy of timescales is seen in mice (Rudelt et al., 2024; reviewed in Li and Wang, 2022). It had long been thought that this gradient of intrinsic activity timescales represented a gradient of intrinsic network timescales created by the gradient of increasingly strong excitatory-to-excitatory connectivity seen from primary sensory to higher sensory to motor and frontal areas in both primates and mice (Elston, 2003; Hsu et al., 2017). Instead, the fast timescale of activity decay upon elimination of thalamic input suggested (to the degree that results from mouse can be extrapolated to primate) that the cortical circuit did not create intrinsic network time constants longer than the intrinsic cellular time constants, and that the long cortical autocorrelation time constants must instead be inherited from thalamic inputs and/or created by the thalamo-cortical loop or by thalamically induced changes in cortical state.

We assumed strong local connectivity in our model, which was needed to explain both the decrease in inhibition with surround suppression and the fact that maximal suppression occurs when surround orientation matches center orientation, even when the latter is non-preferred. Furthermore, the effective synaptic strengths in the network are increased by increasing external input strength (Ahmadian et al., 2013). Although strong recurrent excitatory loops can create patterns of activity with slow decay, this can be prevented if feedback inhibition balances the excitation so that no activity pattern can effectively excite itself (e.g., Murphy and Miller, 2009). Thus, we wish to determine whether the strong local connectivity in our model creates intrinsically long timescales, or whether the “loose balancing” of excitation by inhibition in the model (Ahmadian and Miller, 2021) is sufficient to prevent this, consistent with observations in mouse cortex. Either way, this provides a prediction for primate cortex and an experimental test of our model.

Silencing visual thalamus is equivalent in our model to silencing the feedforward input to the network. We record the activity of a cell for two stimulus sizes, 2° and 10°, each at two contrast levels, high contrast (*C* = 17) and low contrast (*C* = 9). In all cases, the feedforward input is removed at 200 ms. To obtain the activity decay time constant, we fit the decaying activity with an exponential function.

**Figure 11.**
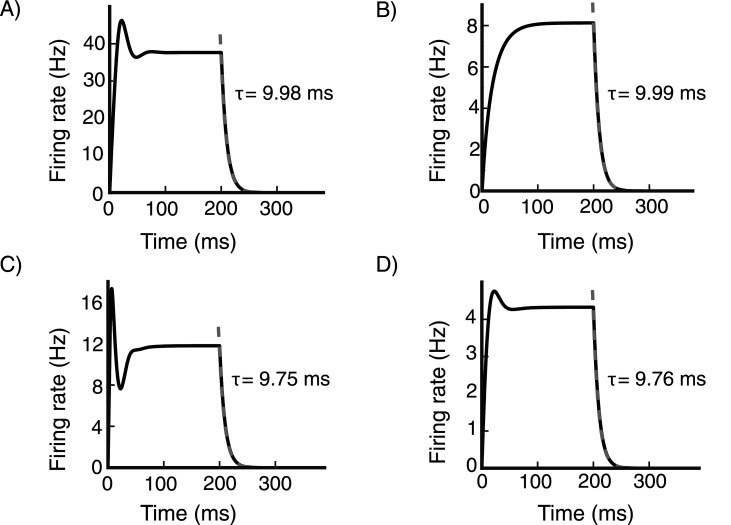
Activity decay time The time response of an excitatory (***E***) cell at a randomly selected grid location (see Section Model, subsection Mode details) for various stimulus conditions. ***A***, and ***B***, 2° size stimulus at high contrast (*C* = 17) and low contrast (*C* = 9) respectively. ***C***, and ***D***, 10° size stimulus at high contrast (*C* = 17) and low contrast (*C* = 9) respectively. The feedforward input is removed at 200 ms. The activity decay time constant is obtained by fitting an exponential function to the decaying activity. The activity decay time constant is roughly independent of the stimulus contrast level and size.

We find that the decay time constant for the excitatory cells in the model is roughly 10 ms independent of stimulus contrast (Fig. 11) as found in V1. We also find that it is roughly independent of the stimulus size (Fig. 11). In particular, For 50 excitatory cells at randomly selected grid locations, the activity decay time constants reported as mean ± s.e.m. are as follows: for a 2° stimulus it is 10.04 ± 0.01 ms at high contrast and 9.99 ± 0.02 ms at low contrast, and for a 10° stimulus it is 9.75 ms at high contrast and 9.76 ms at low contrast (here and below, we omit the s.e.m. if it is <0.005). This decay time is essentially given by the excitatory cells’ time constant, which is 10 ms. Similarly, we find the activity decay time scale for the inhibitory cells in the model to be roughly given by the inhibitory cells’ time constant of 6.67 ms. The activity decay time constants for the inhibitory cells are as follows: for a 2° stimulus it is 6.93 ± 0.01 ms at high contrast and 6.73 ± 0.04 ms at low contrast, and for the 10° stimulus it is 6.69 ± 0.03 ms at high contrast and 6.98 ± 0.05 ms at low contrast.

### Center-surround interactions in a conductance-based spiking model

The rate model that we presented above is able to reproduce multiple center-surround interactions observed in V1. We studied a rate model because it is much simpler to explore parameters and to understand mechanisms in rate models, and they are generally good guides to the behavior of spiking networks when spike synchronization is not involved. However, there is no guarantee that spiking models will show the same behaviors, and differences can arise (e.g., Sanzeni et al., 2020a, 2020b). Therefore, to examine whether the mechanisms we have uncovered will yield the same effects in a more biophysically realistic setting, we now examine a network of conductance-based spiking neurons (see Eq. 7 in Section Model, subsection Model details). To make the model more biologically realistic, we assume the excitatory and the inhibitory cells have spontaneous activity levels of 1.5 Hz and 3 Hz, respectively.

**Figure 12.**
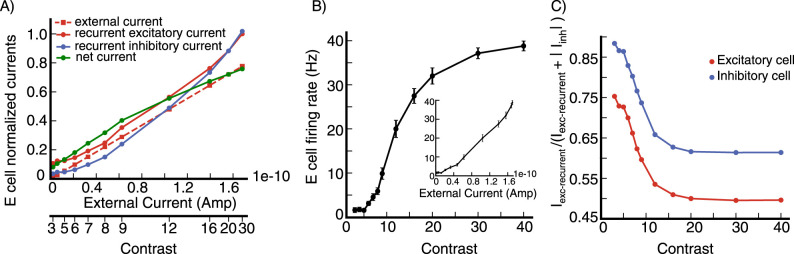
Conductance-based spiking model ***A***, Input currents to an excitatory (***E***) cell at a randomly selected grid location (see Section Model, subsection Model details) vs external input current. The recurrent excitatory current *I*_*exc*−*recurrent*_ = 〈*g*_*E*_ (*R*_*E*_ − *V*)〉_*t*_, the recurrent inhibitory current is the absolute value of *I*_*inh*_ = 〈*g*_*I*_ (*R*_*I*_ − *V*)〉_*t*_ and the external current *I*_*ext*_ = 〈*g*_*in*_ (*R*_*E*_ − *V*)〉_*t*_ where 〈〉_*t*_ denotes time average. The net current is (*I*_*ext*_ + *I*_*exc*−*recurrent*_ + *I*_*inh*_), note that *I*_*inh*_ is negative in the spiking model. All currents are normalized to the peak value of the recurrent excitatory current. Stimulus contrast level corresponding to the external current is shown on the bottom axis. ***B***, Firing rate of the cell in (***A***) vs contrast. ***C***, *I*_*exc*−*recurrent*_/(*I*_*exc*−*recurrent*_ + |*I*_*inh*_|) vs contrast for the cell in (***A***) and an inhibitory cell at the same grid location. In these experiments we use a stimulus with width 2.16° and orientation equal to the cell’s preferred orientation.

We first show how the input currents to a cell and its firing rate change with external drive (Fig. 12*A*,*B*). The network replicates the “loosely balancing” behavior underlying SSN circuit properties (Ahmadian et al., 2013; Rubin et al., 2015; Ahmadian and Miller, 2021), in two respects: (1) The net input current a neuron receives increases rapidly as a function of external current for weak external input, but sublinearly for stronger external input (Fig. 12*A*); (2) The network input becomes increasingly inhibitory with increasing external drive (Fig. 12*C*) (as verified experimentally in Adesnik, 2017).

**Figure 13.**
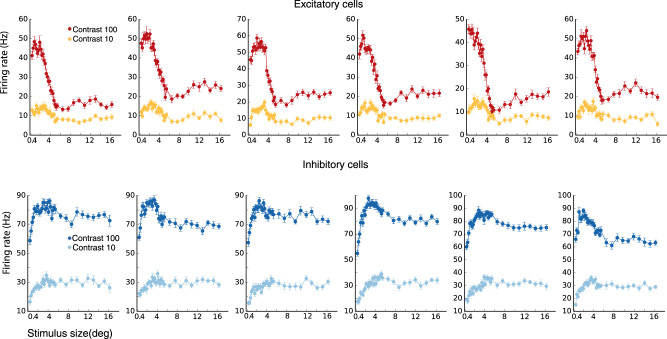
Conductance-based spiking model, surround suppression size tuning curves of 6 excitatory (***E***) cells (top panel) and 6 inhibitory (***I***) cells (lower panel) for two different stimulus contrast levels *C* = 100 and *C* = 10. The error bars are the s.e.m.

We test if our key findings in the rate model also hold in the spiking model. We examine the size tuning curves of the cells in the model. We record the activity of a cell, as we vary the stimulus width from $0.43^{\circ }$ (±1 grid spacing) to $16^{\circ }$. Both excitatory cells and inhibitory cells are surround suppressed, with excitatory cells more strongly surround suppressed than inhibitory cells as seen from the size tuning curves of sample cells (6 E cells and 6 I cells, top and bottom panels, respectively, of Fig. 13). We note that the smallest size stimulus we can use, which corresponds to 2 grid intervals (i.e., ±1 grid interval), already gives responses close to peak response for excitatory cells. With a finer grid, continuous rise of responses from 0 response for size 0 would be seen. We also note that our model cannot reflect the rich variability seen in biological data, because the connectivity and weights in our model are not randomly sampled from probability distributions that give the desired mean dependence on distance, orientation and cell type, but rather are deterministic functions of those quantities.

**Figure 14.**
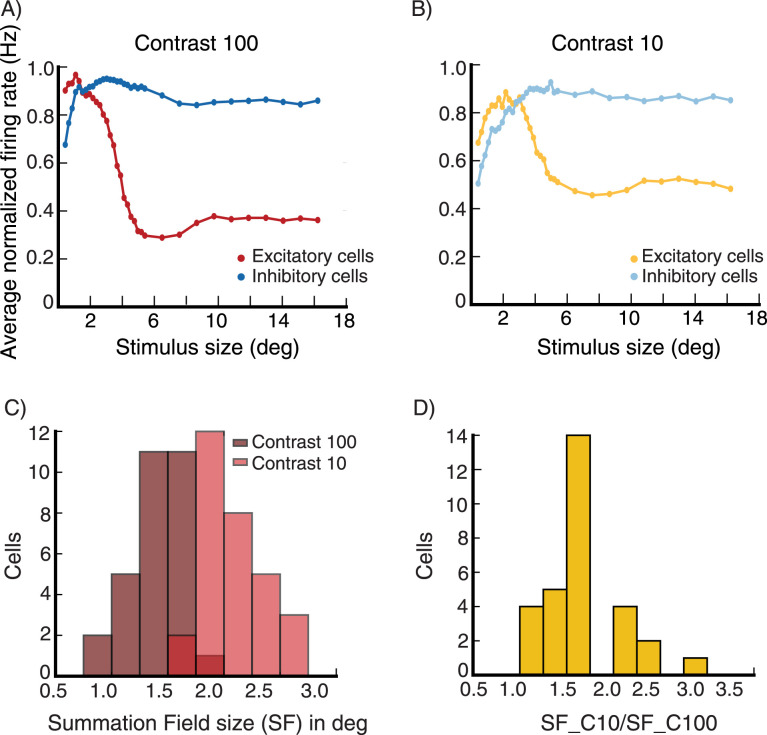
Conductance-based spiking model, surround suppression The average firing rate of 30 excitatory (***E***) cells at randomly selected grid locations (see Section Model, subsection Model details), and of 30 inhibitory (***I***) cells at the same grid locations, after normalizing each cell’s rates so that its peak rate is 1.0, vs. stimulus size at contrast *C* = 100 (***A***) and contrast *C* = 10 (***B***). ***C***, ***D***, Summation Field Sizes. ***C***, The distribution of summation field size of the 30 E cells used to produce panels (***A***) and (***B***) at contrasts *C* = 100 (dark red color) and *C* = 10 (light red color). ***D***, The distribution of the ratio of the summation field sizes in (***C***). The summation field size of all cells, is smaller at the higher contrast stimulus.

Figure 14*A*,*B* shows the average size tuning across 30 E cells and 30 I cells at the same grid locations. The strength of surround suppression increases with increasing stimulus contrast (Figs. 13, 14*A*,*B*). To compute the suppressive index (SI), we fit the cells responses with a double Gaussian function. The mean SI is 0.45 ± 0.07 for the excitatory cells and 0.098 ± 0.054 for the inhibitory cells at *C* = 10, and increases to 0.65 ± 0.09 and 0.14 ± 0.06 at *C* = 100.

The summation field size changes with stimulus contrast level, Figure 14*C* shows the distribution of the summation field sizes of the 30 E cells used to produce Figure 14*A*,*B* for two contrast levels *C* = 100 and *C* = 10. The summation field size decreases with increasing stimulus contrast (Fig. 14*D*).

**Figure 15.**
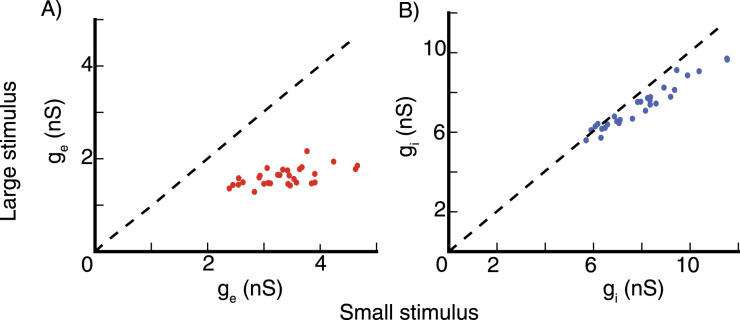
Conductance-based spiking model, surround suppression Excitatory and inhibitory conductances values of the 30 excitatory (***E***) cells used in Figure 14, *g*_*e*_ and *g*_*i*_ are the time average values of the excitatory and inhibitory conductances in Equation 7, respectively. ***A***, Excitatory conductance values of the E cells for a large suppressive stimulus are plotted against their values for a small stimulus size around which the cells respond maximally (we pick the small stimulus size using the method described in Section Results, subsection Surround suppression). ***B***, same as (***A***) but for inhibitory conductances. Stimulus contrast *C* = 100.

To test whether surround suppression in the spiking network is also accompanied by a decrease in excitation and inhibition that the cell receives, as reported by Ozeki et al. (2009), we plot the excitatory conductance values (Fig. 15*A*) and the inhibitory conductance values (Fig. 15*B*) for the same 30 E cells for a large stimulus for which all the cells are suppressed against their values for a small stimulus around which the cells respond maximally (we pick the size of the small stimulus using the same method described in Section Results, subsection Surround suppression). Both excitatory and inhibitory conductances are smaller for the large suppressive stimulus.

**Figure 16.**
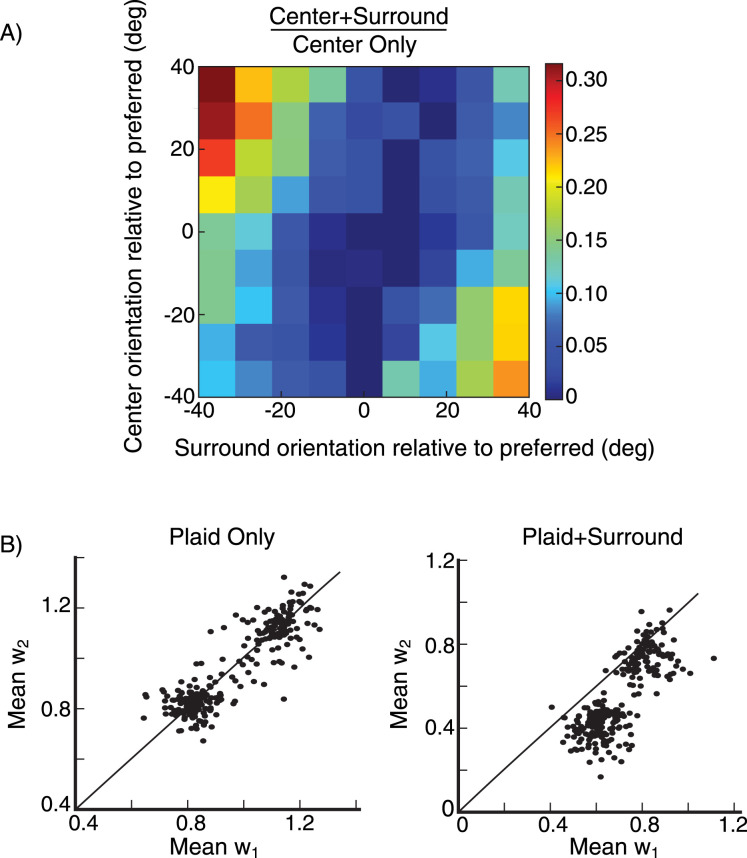
Conductance-based spiking model, surround tuning to the center orientation and feature-specific surround suppression ***A***, Surround tuning to the center orientation, average surround modulation map, the data is from 23 excitatory (***E***) cells at randomly selected grid locations (see Section Model, subsection Model details), same plot as Figure 8*A*. ***B***, Feature-specific surround suppression, the data is from 56 populations centered around 56 randomly selected grid locations (see Section Model, subsection Model details); same plot as Figure 10*A*,*B* however, in the spiking model we tested the effect with fewer plaid angles.

The results for feature-specific surround suppression in the spiking model are qualitatively similar to those in the rate model (compare Fig. 16*B* to Fig. 10*A*,*B*). The results are also similar for matching of surround tuning to the center orientation (compare Fig. 16*A* to Fig. 8*A*). We point out that this is so even though the spiking model, but not the rate model, has noise which can obscure results for low firing rates. Lastly, we did not do any fine tuning of the spiking model parameters to best match the rate model (see Section Model, especially subsection Model details for considerations behind our choices of parameters).

## Discussion

We have demonstrated a simple circuit model that accounts for multiple aspects of contextual modulation: (i) Surround suppression that is accompanied by a decrease in inhibition that a cell receives (Ozeki et al., 2009; Adesnik, 2017), and that, for weaker (lower contrast) stimuli, is weaker (e.g., Cavanaugh et al., 2002; Schwabe et al., 2010) and has larger summation field sizes (e.g., Sceniak et al., 1999); (ii) Surround/center matching: Surround suppression is strongest when surround orientation matches center orientation, regardless of whether or not the center orientation corresponds to the cell’s preferred orientation (Sillito et al., 1995; Shen et al., 2007; Shushruth et al., 2012; Trott and Born, 2015); (iii) Feature-specific surround suppression: When the center stimulus is a plaid—two gratings with different orientations—a surround with orientation matched to one of the center gratings most strongly suppresses the component of response driven by that matched grating (Trott and Born, 2015). Crucial to explaining both the decrease in inhibition and the surround/center matching was particularly strong local connectivity that, for surround/center matching, needed to be broadly tuned for orientation (the decrease in inhibition also requires that excitation alone be unstable, but be stabilized by feedback inhibition, Ozeki et al., 2009). These phenomena arise in a model that also replicates other phenomena previously demonstrated to arise from the SSN mechanism (Rubin et al., 2015; Ahmadian and Miller, 2021). These include, with increasing external input strength, a transition from dominant feedforward drive to dominant recurrent drive, an increasing dominance of inhibition in recurrent input (as observed experimentally: Shao et al., 2013; Adesnik, 2017), and a sublinear growth of net input as a function of external input (and strengthening of surround suppression and shrinking of summation field sizes had been previously shown to arise from the SSN mechanism). We have also shown that, despite the strong recurrent excitation in the model, the network does not create long time scales of activation: on withdrawal of external input, activity decays with the intrinsic single-cell time constant, as observed in experiments (Reinhold et al., 2015; Guo et al., 2017). Finally, we showed that all of these phenomena are replicated in a network of conductance-based spiking neurons with similar architecture, showing that the mechanisms we have uncovered continue to operate in this biophysically more realistic setting.

The especially strong local connectivity that we used may be needed simply to better approximate the density of biological neurons and thus of connections received by neurons, given the much lower cell density in the model compared to the brain. The model makes two notable predictions. First, the local connections must have a much broader tuning for orientation compared to long range connections. This local broad tuning for orientation was especially needed for surround/center matching (Section Results, subsection Surround tuning to the center orientation), and was also suggested by Shushruth et al. (2012) and seen in the axonal projections by Stettler et al. (2002). Second, the ratio of (E→I) to (E→E) synaptic strengths should increase with increasing distance between connected cells. However, although this last condition seemed necessary to achieve the decrease in inhibition with surround suppression, we cannot state with certainty that the phenomena cannot be achieved otherwise.

A defect of the model is that surround/center matching is incomplete, that is, the graph of most suppressive surround orientation vs. center orientation has a slope around 0.5 (see Figs. 8*A*,*C*, 16*A*), whereas experimentally the slope is much closer to 1 (Sillito et al., 1995; Shen et al., 2007; Trott and Born, 2015). The mechanism of surround/center matching (Section Results, subsection Surround tuning to the center orientation) relies on two factors: (1) the local recurrent connectivity is strong enough and broadly tuned enough in orientation, so that the majority of a neuron’s input in response to a center stimulus with non-preferred-orientation comes from nearby cells that prefer that orientation, and (2) the surround input is more narrowly tuned in orientation, centered on cells preferring the same orientation as the center. Thus, the surround/center matching might be improved by taking steps to increase these factors, i.e., increasing the width in orientation and/or strength of the local projections, and increasing the sharpness of tuning of the long-distance projections connecting surround to center.

Trott and Born (2015) argued that surround/center matching might be compatible either with (a) an output-gain model, in which a surround caused a general suppression or normalization of activity in the local circuit regardless of the feature selectivity of neurons, and this suppression was strongest when surround orientation matched center; or (b) an input-gain model, in which a surround specifically suppressed the component of input driven by the center. They argued that the feature-specific suppression they saw with a plaid center stimulus argued for the input-gain model. This suggested a common mechanism underlying surround/center matching and feature-specific suppression, but we have found that the mechanisms differ. Surround/center matching relies on the strong, broadly-tuned local connectivity that causes the largest portion of a cell’s input to come from neighboring cells that prefer the center orientation, along with the specific targeting of long-distance connections to cells of similar preferred orientation. Feature-specific surround suppression does not require broadly tuned local connectivity, but requires the specific targeting of long-distance connections. Indeed historically, we first replicated feature-specific surround suppression in a model that reproduced surround suppression but lacked the broadly-tuned local component of connectivity, we then found that the circuit would not replicate surround/center matching until we introduced this feature of local connectivity (data not shown). Thus, the input-gain model, which is a phenomenological description rather than a mechanistic model, is too simplified in that it lumps together two phenomena—feature-specific surround suppression and surround/center matching—that have differing underlying mechanisms.

The model shows interesting transient behavior at stimulus onset that varies with stimulus size and contrast (Fig. 11). However, these dynamical features require separate study, as we don’t know if they are intrinsic to the mechanisms we are studying: for example, variation of parameters may cause these behaviors to vary without altering the behaviors we are studying here or the mechanisms underlying them. In this work, we do not focus on these dynamical responses. We study only one aspect of dynamics, namely the fast decay time when input is removed. We study this aspect because strong excitatory connectivity, as we use in our model, can create slow decay times if not appropriately balanced by inhibition (e.g., Murphy and Miller, 2009), and we wanted to ensure that our model is consistent with the fast decay times seen biologically.

We have found that a wide range of behaviors can be generated by the basic circuit motif we have studied here. What are the computational functions of these behaviors? One can imagine many advantages to retaining the ability to respond quickly to sudden input changes. The fact that inhibition is reduced in surround suppression can be traced to such suppression being a reduction in “balanced amplification” (Murphy and Miller, 2009), in which a small tilting of the E/I balance toward inhibition leads to large reductions of both excitatory and inhibitory firing; balanced amplification in turn provides a mechanism for amplifying responses without slowing of response. Surround/center matching has been proposed to facilitate detection of orientation discontinuities, such as corners (Sillito et al., 1995). V1 neurons show similar behavior—maximal suppression when center and surround are matched, regardless of a cell’s feature preferences—for a variety of cues including luminance, contrast, color, spatial frequency, and velocity, in addition to orientation (Shen et al., 2007), which was postulated to contribute to cue-invariant shape and object recognition. Surround suppression by an input-gain mechanism, as in feature-selective suppression, has been postulated to play a role in predictive coding (e.g., Lochmann et al., 2012; Aitchison and Lengyel, 2017). Surround suppression has been shown to emerge from Bayesian inference of latent factors underlying the visual image (Coen-Cagli et al., 2015), and the SSN circuit more generally has been shown to implement such inference (Echeveste et al., 2020). We believe that understanding the underlying circuit mechanisms can contribute to clarifying which of these, or other, computations the circuit is carrying out.

## Synthesis

Reviewing Editor: Arvind Kumar, KTH Royal Institute of Technology Decisions are customarily a result of the Reviewing Editor and the peer reviewers coming together and discussing their recommendations until a consensus is reached. When revisions are invited, a fact-based synthesis statement explaining their decision and outlining what is needed to prepare a revision will be listed below. The following reviewer(s) agreed to reveal their identity: Bryan Tripp. Note: If this manuscript was transferred from JNeurosci and a decision was made to accept the manuscript without peer review, a brief statement to this effect will instead be what is listed below.

Synthesis.

The manuscript was reviewed by two reviewers (one had already reviewed it for J. Neurosci.). The reviewers agree that the manuscript makes a noteworthy contribution but there are some limitations. The authors have tuned the parameters of a complex model to fit the data. The interesting result is that E->I connectivity is important but authors do not go into details of under what conditions this kind of connectivity is necessary - a more exhaustive parameter search would be needed for that. However, reviewers think that such an investigation may be beyond the scope of the current manuscript. Still, it is necessary that limitations of the model are discussed. Moreover, reviewers think that the manuscript needs to be presented in a less convoluted manner - the wording at many places needs to be clear and correct. They have made several suggestions that I think will help the authors to revise the text. Besides, there are few aspects of the analysis or figures that need further clarification.

So we invite you to revise the manuscript according to the suggestions of the reviewers (appended below).

Reviewer #1

The paper makes a modest but noteworthy contribution by proposing a unified, biologically plausible model that integrates multiple aspects of surround suppression. However, in its current form, the work appears more focused on parameter tuning of a complex model to match existing findings, rather than demonstrating how these effects emerge naturally from the model. As such, its primary value lies in advancing the line of SSN modelling more than providing novel insights into the biology.

Connectivity:

The most interesting component of the work is the differential E-I connectivity needed to obtain the results (especially, the stronger E->I connectivity compared to E->E). However, while the authors present this differential connectivity as a model prediction, it can also be viewed as a prerequisite that requires experimental validation.

Also, a more comprehensive search of the parameter space is needed to check under which connectivity parameters the surround suppression phenomena (SSP) emerge. Currently, the work is presented as finding some combination of parameters in the SSNs that satisfy the SSP. This is a good computational exercise, and certainly advancing the field of SSN modelling, but it doesn't provide insight into the biological conditions necessary and sufficient for SSP.

Spiking networks:

It is nice that the authors have provided simulations of conductance-based neuronal networks in which the main results hold. It would have been good to see some spike trains or/and spike train statistics to know in which regime these networks are operating.

Writing:

The writing is quite convoluted, both in terms of the general flow of the text and individual sentences with complex or bad structures.

Specific comments:

L. 107: "we find that this requires especially strong local connectivity"

Not sure what "especially strong" means.

L. 126: "... demonstrates that similar mechanisms will operate in the more biological context of a spiking neural ..."

L. 163-169: It's not very useful to mention unpublished work without showing the results (multiple examples throughout the text referring to such unpublished results).

L. 213-215: "With a more realistic density *we don't think* this would be

necessary. For inhibition to decrease with surround suppression, *we also seem to need* the E->I connectivity to become stronger relative to the E->E connectivity ..."

Doesn't read like a scientific style of writing.

L. 216-7: "there is little data on this issue, so this represents a prediction of the model."

L. 221-232: Example of too much tuning to make the SSN model match the results - suggesting that the achievement of the paper is in tuning a rather complex (and biologically realistic) model with many parameter to behave as it should.

L. 242-246: Evidence in favour of the broad tuning of local connections and tighter tuning of long range connections is provided from the literature in monkey and ferret. Yet in the line after the local orientation tuning width of excitatory connections is estimated from the mouse data (while it's not clear if the aforementioned tuning properties of local and long-range connections hold in mice).

L. 345-6: "This corresponds to an annulus inner radius of 2:15 or 1.1 mm which is roughly the span of E-to-I monosynaptic connections"

Would be good to mention the reference for this.

Writing:

L. 404-5: "To compare to experiments, there is now a problem to be solved: as modelers we know the ..."

L. 407: "However experimenters do not know the optimal size, and must choose some size in that vicinity"

L. 420: decease -> decrease

L. 422: "While we cannot exhaustively search all parameters, in our explorations of parameters, we have found ..."

L. 426: "the cells must be strongly enough connected so that .."

L. 439-443: Example of convoluted and non-clear writing.

Also see:

L. 554-557

L. 667-70

L. 614: colloquial rather scientific style of writing / reporting:

"for a 10 stimulus 9.75ms at high contrast and 9.76ms at low contrast (when we do not report it, the s.e.m. is too small).

Strange style of presenting the information:

L. 667: "... qualitatively similar to what we observe in the rate model, Fig. 16B is the same as Fig. 10A,B."

L. 670: "... give a response above the spontaneous activity level, Fig. 16A shows the surround modulation map in the spiking model."

L. 674: "Also, the conductances values are not fine tuned, so a different set of values can for example give larger SI indexes ..."

How are the parameters obtained then? Not clear / explained.

The Discussion especially needs rewriting - multiple examples of bad wording:

L. 706: "despite our necessarily much less dense grid."

L. 707

L. 709: "Another is that the ratio ..."

L. 711-2

L. 716

L. 717

L. 720

L. 745-6

L. 748

L. 755-6

Reviewer #2

It is great to see further development and additional positive results with the SSN.

The model has various limitations that were pointed out by previous reviewers, including unrealistic cell density and ratio of inhibitory to excitatory cells, and lack of diversity of cells. From my perspective it would also be nice to see this model extended to process images rather than driven by idealized responses to a few stimulus features (Eq. 2). However, I don't think any of these limitations should be addressed in this paper. The authors' rationale for their choices is reasonable, and the paper is already quite complex as it is.

Due to this complexity, I think further attention to clarity would make the paper accessible to a wider audience. Anything that can be done to tighten up the wording, even in small ways, would be appreciated. An example suggestion from line 451:

"Cells in V1 are found to be suppressed maximally when the surround stimulus orientation matches the orientation of the center stimulus ..." This could be changed to:

"Cells in V1 are suppressed maximally when the surround stimulus orientation matches the center stimulus orientation ..."

In the current wording it seems to me that "found to be" is already implied by the citations, and that swapping the word order when talking about surround vs. center stimuli slightly obscures what is being compared. Small changes like this could reduce the effort needed to read the paper.

Below are more specific comments related to clarity.

Line 49: "... surround stimulus of a given orientation specifically suppresses that orientation's component of the response to a plaid center stimulus ...". To me this makes it sound as if the other component is not suppressed, but elsewhere in the paper (e.g. Fig 9C) it's clear that the other component is also suppressed, just less so.

Line 181: I don't understand the definition of kj. Is it a vector that's multiplied by x? Should there be a transpose in (1)? Why is sin associated with one dimension and cos with the other?

Line 201: The sigmas seem to have very specific values but the explanation that follows is qualitative. Is there something missing from this explanation?

Line 221: Please explicitly state how the "first condition" (Lines 221-231) constrains J_XY.

Line 240: The J values (e.g. 0.0288) also seem more specific than their rationales. Please explain how they were chosen.

Line 262: Is the RF sigma really 0.09 degrees? How does this relate to summation fields of 1-3 degrees (Figure 14)?

Figure 2: Please comment on the origin of the bump in the right panel.

Line 265: Eq. 2 should be an equation rather than an expression (i.e. please add the variable for external input).

Line 285: Please further explain the last term of the first equation, specifically the part under the square root.

Line 351: Contrast 16.4 is said to produce 80% of max input strength. Is this net input? Does max mean with 100% contrast?

Figure 4: Commas are used strangely in the last two sentences of the caption.

Figure 5: What causes the substantial variation between cells? Distance to other cells with the same orientations?

Figure 6: Please clarify axis units in panels D and E.

Line 455-466: Even after reading this paragraph several times I don't find the explanation intuitive. This sentence in particular doesn't seem to be implied by anything that comes before: "Withdrawal of input from those cells would then cause greater suppression of the firing of the recorded cell than would direct suppression from a surround stimulus at the cell's preferred orientation."

Figure 7: It looks like the red curves are normalized independently but I would prefer that they all had the same scale as the black curves. As it is, I find it hard to visualize what the cell is doing overall.

Line 516: Please state at this point in the text how the two gratings differ from each other. It only gradually became clear to me as I read the paragraph.

Line 524: I found some of this wording confusing. " ... and measure the new values of w1 and w2" This is not really a measurement but the result of an optimization, so a better word might be "find". "We repeat the same procedure as we rotate the plaid ..." Somehow this wording also threw me off. Suggest something like this: "We then repeatedly rotate the plaid stimulus in increments of 10 degrees, and find new w1 and w2 for each plaid orientation ..."

Figure 9A: What causes the large difference between the amplitudes of the P1 and P2 population responses?

Figure 14: "... after normalizing each cell's rates so that its peak rate is 1.0 ..." Please clarify as the peak rates aren't 1.0 in the figure.

Line 664: Please use the updated section name here.

Figure 15: What are g_e and g_i exactly? Can you use the same variable names as in Eq. 7?

Figure 16: Why are there two clusters in panel B?

Line 713: Please check the wording in the abstract for consistency with this limitation of the model.

Line 725: It would be nice to break up this long sentence.

Line 740: I've seen the word "phenomenological" used in this way before, but I don't think it's correct. My understanding is that it relates to conscious experience. "Empirical" might be better here. https://en.wikipedia.org/wiki/Phenomenology_(philosophy)
